# The Role of Strontium in CeNiO_3_ Nano-Crystalline Perovskites for Greenhouse Gas Mitigation to Produce Syngas

**DOI:** 10.3390/molecules27020356

**Published:** 2022-01-06

**Authors:** Naushad Ahmad, Rizwan Wahab, Salim Manoharadas, Basel F. Alrayes, Manawwer Alam, Fahad A. Alharthi

**Affiliations:** 1 Department of Chemistry, College of Science, King Saud University, P.O. Box 2454, Riyadh 11451, Saudi Arabia; maalam@ksu.edu.sa (M.A.); fharthi@ksu.edu.sa (F.A.A.); 2 Department of Zoology, College of Science, King Saud University, P.O. Box 2454, Riyadh 11451, Saudi Arabia; rwahab@ksu.edu.sa; 3 Central Laboratory, Department of Botany and Microbiology, College of Science, King Saud University, P.O. Box 2454, Riyadh 11451, Saudi Arabia; smanoharadas@ksu.edu.sa; 4 Central Laboratory, College of Science, King Saud University, P.O. Box 2454, Riyadh 11451, Saudi Arabia; bfalrayes@ksu.edu.sa

**Keywords:** perovskites, Sr, ceria, H_2_, syngas, carbon deposition

## Abstract

The transition metal-based catalysts for the elimination of greenhouse gases via methane reforming using carbon dioxide are directly or indirectly associated with their distinguishing characteristics such as well-dispersed metal nanoparticles, a higher number of reducible species, suitable metal–support interaction, and high specific surface area. This work presents the insight into catalytic performance as well as catalyst stability of Ce_x_Sr_1−x_NiO_3_ (x = 0.6–1) nanocrystalline perovskites for the production of hydrogen via methane reforming using carbon dioxide. Strontium incorporation enhances specific surface area, the number of reducible species, and nickel dispersion. The catalytic performance results show that CeNiO_3_ demonstrated higher initial CH_4_ (54.3%) and CO_2_ (64.8%) conversions, which dropped down to 13.1 and 19.2% (CH_4_ conversions) and 26.3 and 32.5% (CO_2_ conversions) for Ce_0.8_Sr_0.2_NiO_3_ and Ce_0.6_Sr_0.4_NiO_3_, respectively. This drop in catalytic conversions post strontium addition is concomitant with strontium carbonate covering nickel active sites. Moreover, from the durability results, it is obvious that CeNiO_3_ exhibited deactivation, whereas no deactivation was observed for Ce_0.8_Sr_0.2_NiO_3_ and Ce_0.6_Sr_0.4_NiO_3_. Carbon deposition during the reaction is mainly responsible for catalyst deactivation, and this is further established by characterizing spent catalysts.

## 1. Introduction

Among the various well-established CO_2_ conversion processes, such as electrochemical catalysis, photocatalysis, and thermal catalysis, methane reforming using carbon dioxide, commonly known as dry reforming of methane (DRM), has recently attracted scientists primarily because DRM converts major greenhouse gases, i.e., carbon dioxide and methane, to synthesize hydrogen and carbon monoxide, also called synthesis gas, which is further utilized to produce liquid hydrocarbons [1,2,3,4,5,6,7,8]. Hence, the DRM process not only plays a role in greenhouse gas mitigation and thus serves as a cause of climate change, but also generates synthesis gas, a mixture of equimolar hydrogen and carbon monoxide suitable for the production of hydrocarbon via the well-known Fischer–Tropsch synthesis process [9,10,11,12,13]. It is well established that the proper choice and suitable design of a catalyst plays significant role in catalytic activity and stability during DRM [14,15,16]. Both noble metal-based and transition metal-based catalysts have been reported for DRM, but noble metal-based catalysts are expensive and less abundant despite their excellent catalytic activities [8]. Therefore, transition metal-based catalysts serve as an efficient alternative for DRM. Among transition metal-based catalysts, nickel (Ni)- and cobalt (Co)-based catalysts are most investigated for DRM due to the fact that these catalysts are cost-effective, more abundant, and offer quick turnover rates [17,18,19,20]. The major challenge related to Ni-based catalysts is their deactivation during DRM, associated with sintering (which leads to loss of active metal surface area) and carbon deposition [21,22,23].

In general, the metallic nanoparticles supported on basic oxide supports and/or promoters, such as Sr^2+^, La_2_O_3_, and CeO_2_, etc., demonstrate better catalytic activity and benefit CO_2_ chemisorption compared to acidic supports. Ceria and modified-ceria supports have been found to play a promising role in the endothermic DRM process due to the decisive factors associated with these supports, including their high oxygen storage capacity and/or oxygen vacancies, which not only activate CO_2_ but also help to gasify carbon deposited during DRM and their basicity, which further promotes CO_2_ adsorption [15,23,24,25]. These characteristic features, when combined with tiny (nano)-metallic particles, exhibit better catalytic efficiency, lower the reaction temperature, and promote the water–gas-shift reaction. For instance, Makri et al. [26] synthesized 5 wt% Ni/Ce_1−x_MxO_2−__δ_ (M = Zr^4+^, Pr^3+^, x = 0.2 and 0.5) catalysts by citrate route sol-gel and studied the influence of chemical composition on nickel particle size, which in turn influenced the different kind of carbon deposition under dry reforming reaction conditions.

In addition to the metallic nanoparticle-supported oxides, nickel-based double-layered hydroxides (LDHs) have shown excellent catalytic performances towards DRM reaction [27,28,29,30]. Modification of double-layered hydroxides with Y, Zr, and Ce as dopants or promotors significantly improves CH_4_ and CO_2_ conversions, lowers carbon deposition, and limits side reactions [28,29,30].

Although supported catalysts and LDHs have excellent catalytic performance in the DRM process, they eventually deactivate due to rapid growth of randomly sized impregnated nanoparticles, which assist the carbon deposition and blockage of active sites at higher temperature. To sort out the coalescence and coking deposition, well-defined crystal structures such as perovskite (ABO_3_) and pyrochlore (A_2_B_2_O_7_) are more suitable candidates for the DRM process because they produce uniformly distributed metallic nanoparticles under a reducing atmosphere (B/AO_x_).

Ni-based perovskites are thermally stable and offer high metal dispersion, which leads to their excellent performance in DRM [31,32]. The partial substitution at the A or B sites in ABO_3_ has a distinguished influence for the development of the perovskite phase, especially when substitutions are performed with the metal ions that have a different valence state—for example, substitution of the A trivalence site by bivalence or tetravalence ions (Sr^2+^, Ca^2+^, or Ce^4+^) would relatively improve the size and dispersion of exsolved active metal nanoparticles and increase the synergetic interaction between the active metals and the derived support [33,34,35,36,37,38]. These derived factors are useful for better catalytic activity, more carbon, and metal sintering resistance.

The investigation of the role of supported La_x_NiO_y_/MgAl_2_O_4_ and bulk La_x_NiO_y_ catalysts for dry methane reforming showed that the supported La_x_NiO_y_/MgAl_2_O_4_ catalysts exhibited improved catalytic activity and catalyst durability for over 65 h time-on-stream with less carbon deposition, which was attributed to their larger specific surface areas and higher nickel dispersions. It was also found by XRD analysis of spent La_x_NiO_y_/MgAl_2_O_4_ catalysts that metallic nickel active sites were intact even after 65 h of reaction [39]. Ruocco et al. [40] studied the influence of ternary perovskites AZrRuO_3_ (A = Ca, Ba, Sr), their synthesis technique, and operating conditions such as reaction temperature and gas hourly space velocity for the dry methane-reforming reaction. They discovered that ternary perovskite with Sr (SrZrRuO_3_) demonstrated higher activity and durability than the rest of the perovskites [40]. Utilizing perovskite as a support and subsequent deposition of nickel and cobalt to obtain bimetallic catalysts (Ni-Co/La_2_O_3_-LaFeO_3_) to analyze their activity and stability during dry methane reforming was reported by Wang et al. [41]. The crystalline structure of the perovskite, after a suitable amount of Co was added to the bimetallic catalyst, resulted in enhanced catalytic activity and stability with less carbon formation during the reforming reaction.

This research article demonstrates the impact of strontium incorporation into cerium- and nickel-based nanocrystalline perovskites (CeNiO_3_) in dry and/or CO_2_ reforming of methane. The catalytic activity performances are specifically elucidated in relation to their characterizations before and after reaction. Overall, the study is focused on exploring the insights of cerium replacement with strontium and the influence of this replacement on catalytic conversions and durability under reforming conditions.

## 2. Results

### 2.1. Characterization of Fresh Catalysts

#### 2.1.1. Thermal Decomposition of Precursors

**Figure 1:**
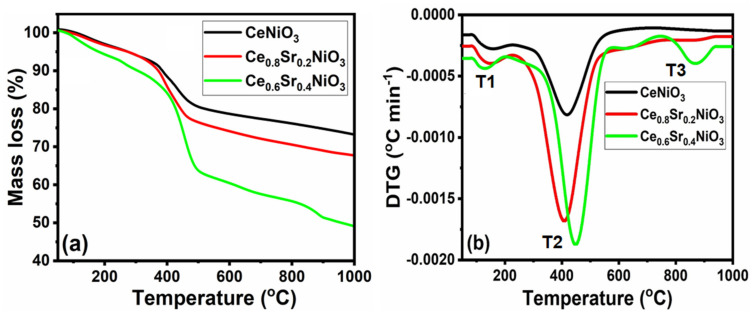
TG−DTG curves versus temperature of Ce_x_Sr_1−x_NiO_3_ (x = 0.6–1) perovskites.

Thermal decomposition curves, including thermogravimetric (TG) and their differentials (DTG) of the precursors as a measure of their calcination temperatures, are shown in Figure 1. It can be seen from Figure 1 that each precursor, a gel-like mixture of metal nitrates and glycine, decomposed differently and both CeNiO_3_ and Ce_0.8_Sr_0.2_NiO_3_ demonstrated two-step decomposition, whereas three-step decomposition was observed for Ce_0.6_Sr_0.4_NiO_3_. Overall, the decomposition temperatures of CeNiO_3_ were relatively higher than strontium-incorporated Ce_0.8_Sr_0.2_NiO_3_ and Ce_0.6_Sr_0.4_NiO_3_. The onset temperature for initial weight loss (first-step decomposition) of ~8%, in the case of CeNiO_3_, was found to be ~75 °C; weight loss then reached its maximum at ~150 °C, and finally finished at ~300 °C. This first-step decomposition was assigned to the removal of the adsorbed water and/or release of adsorbed gases. The second-step decomposition exhibited a weight loss of ~21% observed in the temperature range of 300–550 °C (with peak maximum at 420 °C), which could be ascribed to the decomposition and/or combustion of organic matter. Despite showing an almost similar decomposition trend, the DTG peak temperatures for first- and second-step decomposition for Ce_0.8_Sr_0.2_NiO_3_ dropped slightly down to 145 and 410 °C, respectively.

In contrast, Ce_0.6_Sr_0.4_NiO_3_ demonstrated three-step decomposition, and the onset temperature for initial weight loss was observed to be 130 °C, which was attributed to the release of physically adsorbed water and/or gases. Second- and third-step decompositions, occurring at 450 and 870 °C, elucidated the combustion of organic matter present in the precursor.

#### 2.1.2. X-ray Diffraction (XRD)

**Figure 2:**
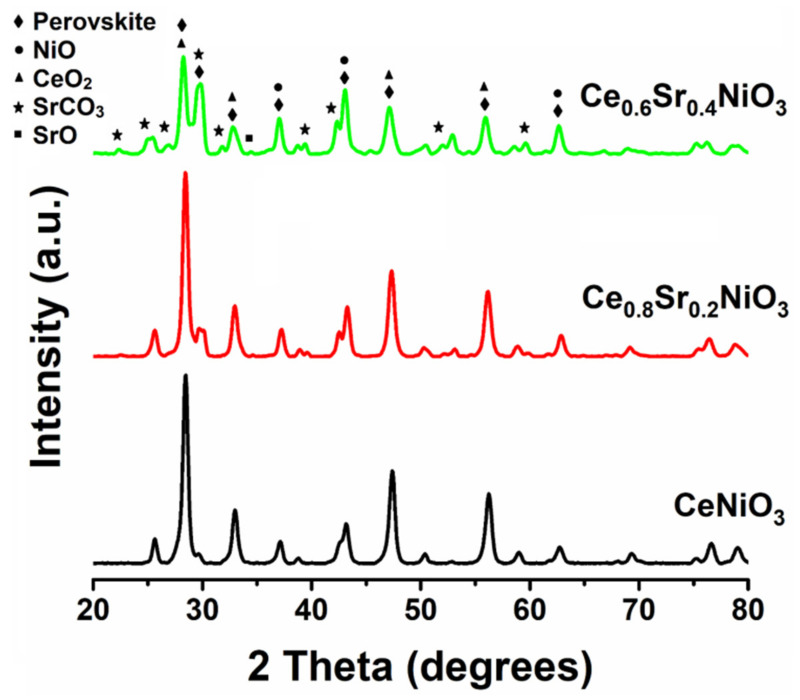
XRD patterns of Ce_x_Sr_1−x_NiO_3_ (x = 0.6–1) perovskites.

Figure 2 presents the XRD patterns of the as-prepared nanocrystalline perovskites with and without the incorporation. MDI Jade^®^ software was used to analyze XRD data. The data analysis shows that a significant number of peaks corresponding to CeNiO_3_ were recorded [42,43,44,45], whereas diffraction peaks detected at 2θ values of 37.3°, 43.3°, and 62.9° were related to cubic NiO. Moreover, remaining peaks were assigned to oxides and carbonates of strontium, as labeled in Figure 2 [46].

#### 2.1.3. Textural Properties and Elemental Analysis

**Figure 3:**
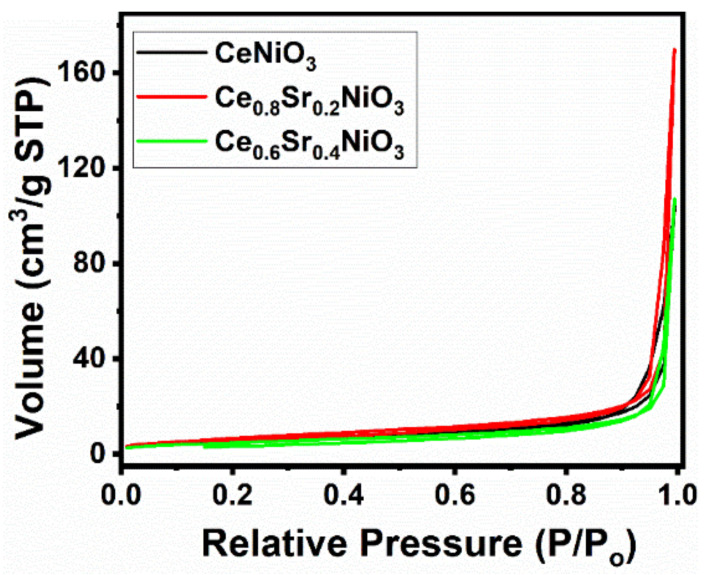
N_2_ adsorption–desorption isotherms of Ce_x_Sr_1−x_NiO_3_ (x = 0.6–1) perovskites.

Figure 3 shows the N_2_ adsorption–desorption isotherms and pore-size distributions of air-calcined precursors (700 °C, 5 h), and their BET surface areas and pore parameters are summarized in Table 1. Figure 3 shows that all of the samples displayed a type II isotherm with a H3 hysteresis loop in the relative pressure (P/P_0_) range of 0.8–1.0, indicating that these samples possessed macro-pores. The specific surface areas observed (17.3–25.6 m^2^/g) are in accordance with the literature, except that these values were slightly higher than the reported specific surface area, i.e., below 10 m^2^/g for similar types of materials [47]. The surface area of Ce_0.8_Sr_0.2_NiO_3_ catalyst was higher (25.6 m^2^/g) than that of the other fabricated catalysts. However, no clear trend as a function of Sr substitution extent was observed. Although Sr doping did not much affect the surface area, it greatly influenced the pore size and pore volume; the highest values were found for x = 0.2 and the lowest for x = 0.0. The increase in pore volume post Sr addition indicates that Sr could play a role in influencing the catalytic activity, which is discussed in Section 2.2 and Section 3. Furthermore, the elemental analysis of as-synthesized catalysts revealed that CeNiO_3_ catalyst exhibited an Ni/Ce ratio of 1, and hence, both Ni and Ce had similar compositions as measured from ICP-OES (Table 1). The chemical compositions of Sr-incorporated catalysts show that Ce was replaced with Sr.

#### 2.1.4. Morphological Study (TEM) of Fresh and Reduced Perovskites

**Figure 4:**
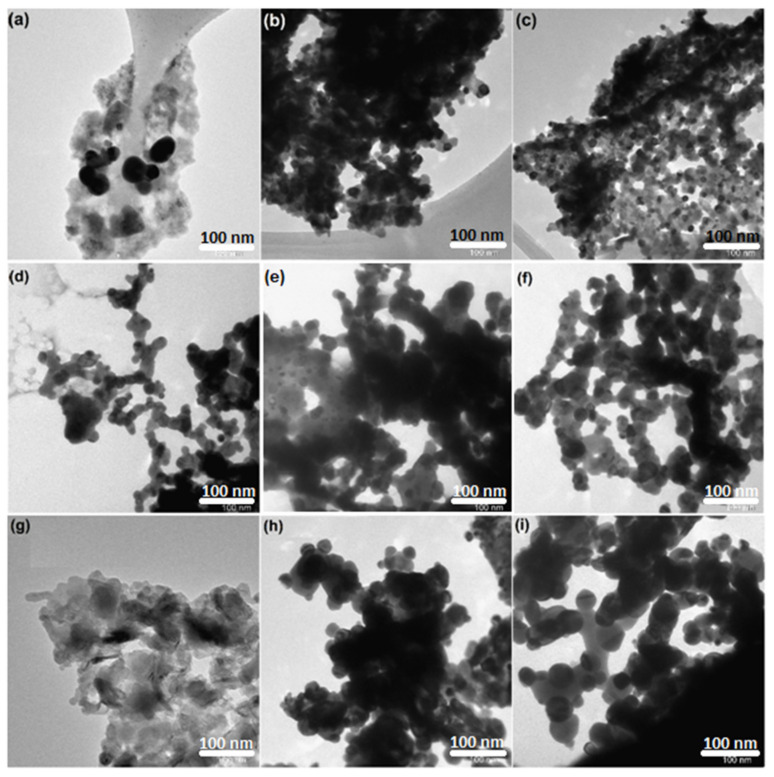
TEM images of Ce_x_Sr_1−x_NiO_3_ (x = 0.6–1) perovskites; fresh catalysts: (**a**) CeNiO_3_, (**b**) Ce_0.8_Sr_0.2_NiO_3_, (**c**) Ce_0.6_Sr_0.4_NiO_3_; reduced catalysts: (**d**) CeNiO_3_, (**e**) Ce_0.8_Sr_0.2_NiO_3_, (**f**) Ce_0.6_Sr_0.4_NiO_3_; spent catalysts: (**g**) CeNiO_3_, (**h**) Ce_0.8_Sr_0.2_NiO_3_, (**i**) Ce_0.6_Sr_0.4_NiO_3_.

Transmission electron microscopy (TEM) was utilized to analyze the morphology of the fresh, reduced, and used nanocrystalline perovskite catalysts, and microscopic images of all the catalysts before and after reaction are shown in Figure 4. ImageJ^®^ software was used for image processing and data analysis. The particles were observed to be spherical in shape, and the average particle sizes were between 5–34, 12–27, and 14–25 nm for CeNiO_3_, Ce_0.8_Sr_0.2_NiO_3_, and Ce_0.6_Sr_0.4_NiO_3_ fresh catalysts, respectively. It is interesting to note that insignificant sintering was observed for reduced catalysts, and particle sizes were 8–45, 15–42, and 18–40 nm for CeNiO_3_-, Ce_0.8_Sr_0.2_NiO_3_-, and Ce_0.6_Sr_0.4_NiO_3_- reduced catalysts, respectively.

#### 2.1.5. Temperature-Programmed Reduction (TPR)

**Figure 5:**
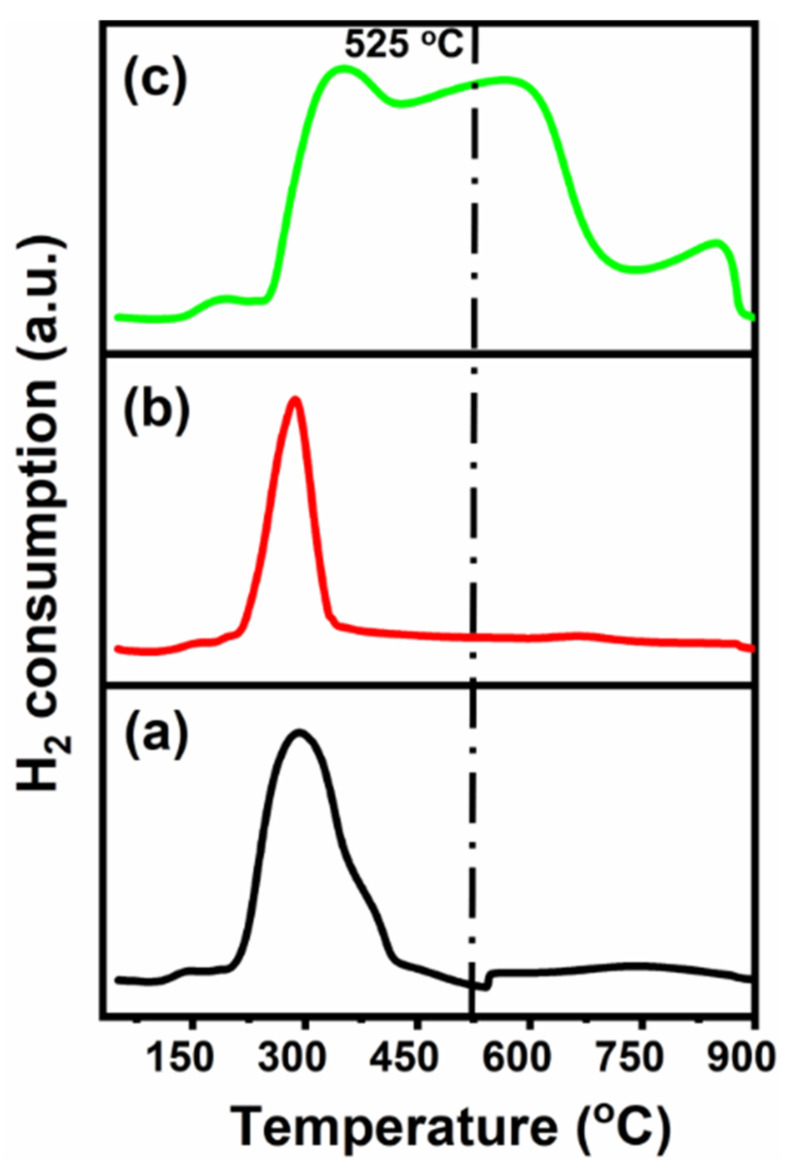
H_2_-TPR profiles of (**a**) CeNiO_3_, (**b**) Ce_0.8_Sr_0.2_NiO_3_, and (**c**) Ce_0.6_Sr_0.4_NiO_3_ perovskites.

TPR is mainly utilized to examine the reducibility, the metal–support interaction, and to find the activation and/or reduction temperature that results in metallic particles required for the catalytic reforming reaction. Figure 5 presents the reduction profiles, indicating the variations in metal–support interaction and/or the reducibility of CeNiO_3_ post Sr addition. The reduction profiles can be divided into two regions (region I < 525 and region II > 525 °C). The small reduction peak in region I below 220 °C in each catalyst is related to the reduction of weakly interacting nickel oxide species, whereas the reduction peaks appearing between 220 and 525 °C correspond to two-step reduction of Ni^3+^ to Ni^2+^ and Ni^2+^ to Ni^0^ [44,48]. The reduction profiles in region II are ascribed to either the reduction of ceria and/or the reduction of perovskite to metallic nickel at higher temperature [49]. CeNiO_3_- and Sr-incorporated catalysts reduce according to the reactions below:

CeNiO_3_ + H_2_ = Ni^0^ + CeO_2_ + H_2_O (1)

Ce_x_Sr_1−x_NiO_3_ + H_2_ = Ni^0^ + (1 − x)SrO_1−x_ + xCeO_2_ + H_2_O (2)

There were no significant change in the reduction peak temperatures (~290 °C) of CeNiO_3_ or Ce_0.8_Sr_0.2_NiO_3_; however, the peak intensity of Ce_0.8_Sr_0.2_NiO_3_ reduced to one third of CeNiO_3_, indicating the suppression of a number of reducible species post Sr addition. This trend completely changed when Sr was further added (Ce_0.6_Sr_0.4_NiO_3_), and more species reducible at higher temperatures were observed. It is reported that the addition of Sr to a supported Ni catalyst enhanced metal–support interaction and new peaks were found at higher temperature, which is in agreement with the TPR results of Ce_0.6_Sr_0.4_NiO_3_ but is contradictory to the Ce_0.8_Sr_0.2_NiO_3_ reduction profile, where a small shoulder was observed in region II. The enhanced reducibility and/or easier reduction of Ce_0.8_Sr_0.2_NiO_3_ can be attributed to a possible lowering of the valence state of Ni cations incorporated into the oxide. The role of these findings in influencing the catalytic activity is discussed in Section 2.2.

### 2.2. Catalytic Performances

**Figure 6:**
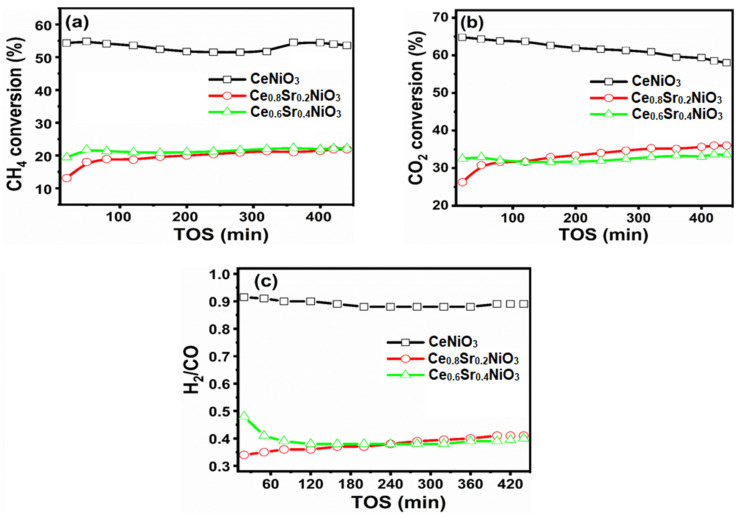
(**a**) CH_4_ conversion, (**b**) CO_2_ conversion, and (**c**) H_2_/CO ratios versus time-on-stream (TOS) of Ce_x_Sr_1−x_NiO_3_ (x = 0.6–1) perovskites.

As-synthesized nanocrystalline Ce_x_Sr_1−x_NiO_3_ (x = 0.6–1) perovskites were studied for their performance in dry methane-reforming reaction at 700 °C. Prior to the dry-reforming reaction, all the catalysts were subjected to reduction/activation using hydrogen as a reducing gas to generate the required metallic nickel crystallites as active sites. Figure 6a,b shows methane (CH_4_) and carbon dioxide (CO_2_) conversions versus time-on-stream. It can be seen from Figure 5a that CeNiO_3_ demonstrated a higher CH_4_ conversion than Sr-incorporated catalysts. CeNiO_3_ exhibited an initial CH_4_ conversion of 54.3% and deactivated down to reach 50.1% after 7 h time-on-stream, leading to a deactivation factor of 7.7% (Table 1). The deactivation factor based on first-order kinetics also showed the same deactivation trends, as shown in Table 1 [50,51]. The impact of incorporating Sr into CeNiO_3_ over initial CH_4_ conversion was obvious, and decreased from 54.3% (CeNiO_3_) to 13.1% (Ce_0.8_Sr_0.2_NiO_3_) and 19.4% (Ce_0.6_Sr_0.4_NiO_3_) when x changed from 1 to 0.6. The loss in catalytic activity, in the case of Sr-incorporated catalysts, can be attributed to either nickel species agglomeration facilitated by Sr addition after the reduction or the covering of nickel active sites with Sr. Based on the TEM images of reduced catalysts, it is obvious that no significant sintering was observed after reduction; hence, the main reason behind the lower activity of Sr-incorporated catalysts might be nickel active sites covered with Sr. Interestingly, no deactivation was observed for strontium-incorporated perovskites (Ce_0.8_Sr_0.2_NiO_3_ and Ce_0.6_Sr_0.4_NiO_3_) and hence, deactivation factors were negative in these catalysts (Table 1). Moreover, conversions increased for the initial 90 min before becoming constant for strontium-incorporated perovskites, which is associated with the reduction of nickel species remaining oxidized even after activation under hydrogen atmosphere prior to reaction. The catalyst’s deactivation and associated factors behind deactivation are discussed in Section 3. Like Figure 6a,b shows a similar trend for CO_2_ conversions as a function of time. CO_2_ conversions of 64.8, 26.3, and 32.5% were exhibited by CeNiO_3_, Ce_0.8_Sr_0.2_NiO_3_, and Ce_0.6_Sr_0.4_NiO_3_, respectively, which eventually reached final CO_2_ conversions of 58, 36, and 33.6%, respectively. It is noteworthy from Figure 6 that CO_2_ conversions were higher than CH_4_ conversions. This finding leads to the fact that some side reactions were simultaneously taking place, such as the reverse water–gas-shift reaction (CO_2_ + H_2_ → CO + H_2_O) producing H_2_/CO molar ratios lower than unity, i.e., H_2_/CO = 1.0, since hydrogen was consuming CO_2_ (Figure 6c), as well as the Boudouard reaction and CO disproportionation reaction (2CO → CO_2_ + C). A dedicated section discusses the catalytic performance results in relation to their characterizations in Section 3.

### 2.3. Characterization of Spent Perovskites

The post dry reforming perovskites were further analyzed using different characterizations to explain the catalytic activity and/or stability results. Among the tested catalysts, only CeNiO_3_ deactivated over time, whereas Sr-incorporated catalysts showed increasing CO_2_ and CH_4_ conversions. Moreover, CO_2_ conversions higher than CH_4_ conversions (Figure 6) paved the way for side reactions, including reverse CO disproportionation and reverse water–gas-shift reaction. These post dry-reforming catalysts were characterized by temperature-programmed oxidation (TPO) and transition electron microscope (TEM) to explore the modifications experienced by perovskites during the dry-reforming reaction.

#### 2.3.1. Temperature-Programmed Oxidation (TPO)

**Figure 7:**
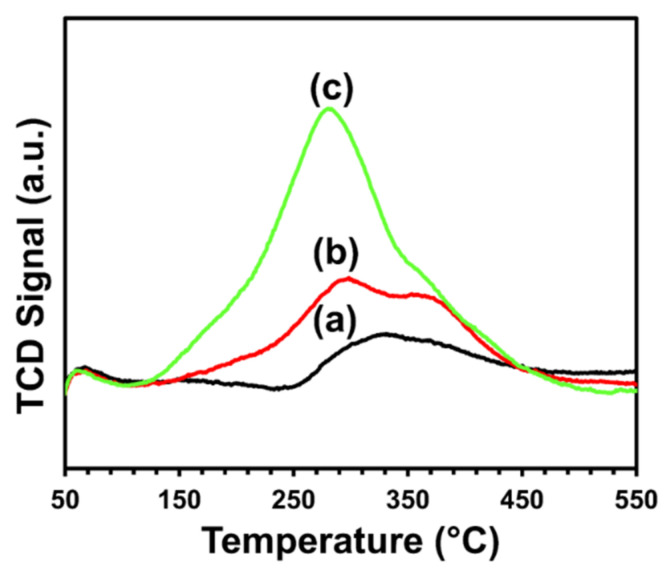
TPO profiles of (**a**) CeNiO_3_, (**b**) Ce_0.8_Sr_0.2_NiO_3_, and (**c**) Ce_0.6_Sr_0.4_NiO_3_ perovskites.

The possibility of carbon formation over the catalyst’s surface post dry-reforming reaction was demonstrated by TPO analysis, as shown in Figure 7. It is obvious that one broad peak was displayed in the temperature range of 110 to 500 °C for each catalyst. The TPO peak maxima were 285, 300, and 335 °C for Ce_0.6_Sr_0.4_NiO_3_, Ce_0.8_Sr_0.2_NiO_3_, and CeNiO_3_, respectively. These peak maxima suggest the formation of less-reactive surface carbides and/or polymeric carbon species during dry methane-reforming reaction, as reported earlier in the literature [52,53]. The hydrogenation degree of surface carbon species is related to TPO peak temperature, and these surface carbon species transform into graphitic carbon as TPO peak temperature moves towards higher temperatures. Furthermore, the peak temperature implies that the interaction between polymeric carbon species and catalyst surface varies and becomes weaker after Sr incorporation, as exhibited by the decrease in TPO peak maxima from 335 to 285 °C for CeNiO_3_ and Ce_0.6_Sr_0.4_NiO_3_. It is noteworthy that the carbon deposition over perovskite samples in this work was significantly lower than scandium- and magnesium-modified Ni- and Co-based bimetallic catalysts supported over an expensive SBA-15 support [3] and strontium-promoted Ni and Co-Al_2_O_3_ catalysts [4]. It can be concluded that cerium oxide and strontium incorporation influence the formation of carbon, carbon suppression, and carbon interaction with the catalyst surface.

#### 2.3.2. Transition Electron Microscopy (TEM) and Elemental Analysis

In order to gain insight into the morphological changes and/or formation of deactivating coke over the surface of spent catalysts, TEM microscopic images were recorded post dry-reforming reaction. From the TEM images of spent catalysts in Figure 4g–i, particle sizes were found to be 10–70, 18–50, and 20–67 nm for CeNiO_3_, Ce_0.8_Sr_0.2_NiO_3_, and Ce_0.6_Sr_0.4_NiO_3_, respectively. The deposition of carbon over the catalysts’ surface and obvious catalyst particle agglomeration were also evident, which suggests the contribution of sintering towards the deactivation of CeNiO_3_. No deactivation, in the case of Sr-incorporated perovskites, was observed even after carbon deposition and particle agglomeration. These findings are further elaborated below in Section 3. Moreover, the elemental analysis of spent catalysts demonstrated that no variations in the chemical compositions of the catalyst were observed during the reaction.

## 3. Discussion

Prior to the dry methane-reforming reaction study, as-prepared nanocrystalline perovskites were characterized to envisage their activity performance during reforming reaction. Thermogravimetric analysis results, i.e., TG-DTG analysis data, illustrate the conversion of precursors into perovskites post calcination treatment at temperatures demonstrated by DTG (Figure 1 and Table S1). XRD diffraction profiles of as-synthesized catalysts (Figure 2) revealed the presence of nickel oxide, perovskite structure, and cerium dioxide along with carbonates and oxides of strontium. The formation of macroporous higher-specific-surface-area perovskite, e.g., 25.6 m^2^/g for Ce_0.8_Sr_0.2_NiO_3_, was obtained from nitrogen adsorption–desorption isotherms (Figure 3 and Table 1). TEM demonstrated (Figure 4) spherical-shaped particles with various sizes with and without strontium incorporation. The reduction profiles using TPR (Figure 5) exhibited easier reduction of oxides in CeNiO_3_ and Ce_0.8_Sr_0.2_NiO_3_, whereas stronger metal–support interaction and difficult-to-reduce oxides were formed in Ce_0.6_Sr_0.4_NiO_3_. Pre-reforming reaction characterization results indicate a higher number of reducible species, larger specific surface area, and a wide range of spherically shaped nanoparticles post strontium incorporation. Hence, it was expected that Sr-incorporated perovskite, such as Ce_0.8_Sr_0.2_NiO_3_, would potentially outperform the rest of the catalysts, but Figure 6 shows lower activity of this catalyst. The mechanism associated with dry methane reforming requires reactant adsorption over the active sites of the catalyst as an initial step. The reactants then dissociate and finally react with each other to form products that eventually leave the catalyst surface following desorption [4]. Nickel, in its metallic form, serves as the active site for the adsorption of CH_4_ [3]. Catalytic activity results reveal the loss in activity in terms of both CH_4_ and CO_2_ conversions post Sr incorporation into CeNiO_3_, which could be ascribed to the loss of active sites due to metal particle agglomeration, the presence of unreduced nickel oxides post activation, and/or active site coverage with carbonates or oxides of strontium [4,7]. TEM data show the extent of metal agglomeration or sintering prior to, during, and post reforming reaction, and it is evident that the degree of sintering remained the same and can be ruled out as a deactivation factor contributing to the lower activity of Sr-incorporated catalysts. Furthermore, the presence of strontium oxide and strontium carbonates, as evidenced by XRD, substantiate the hypothesis that coverage of nickel active sites by strontium species causes loss of catalytic activity in Sr-incorporated catalysts.

**Figure 8:**
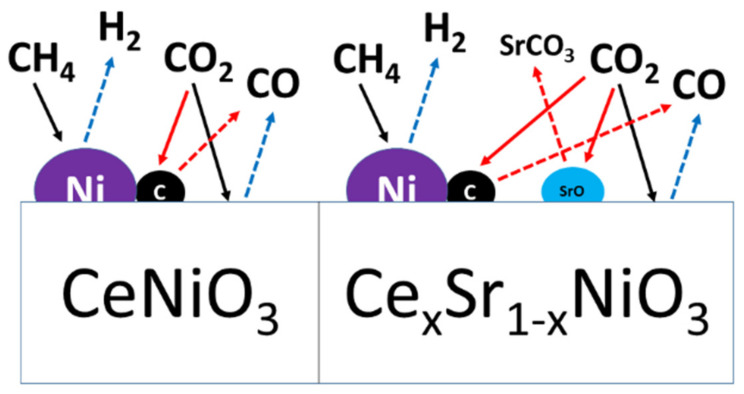
Schematic diagram of the reaction mechanism over Sr-free and Sr-incorporated perovskites.

The activity results versus time-on-stream as a function of catalyst stability are shown in Figure 6. Despite higher activity, CeNiO_3_ perovskite deactivated over time, which is attributed to carbon formation as well as active metal sintering. These observations are substantiated by TEM and TPO characterization results of used catalysts. It is interesting to note that strontium-incorporated perovskites did not show any deactivation despite carbon deposition and sintering. The degree of agglomeration or sintering remained the same for all perovskites during the dry methane-reforming reaction. This suggests that methane decomposition [3], a prevalent DRM side reaction at higher reaction temperature, is the main source of carbon deposition causing catalyst deactivation, in accordance with TPO peak maxima—i.e., a higher temperature (335 °C) is required to gasify carbon formed over the surface of CeNiO_3_ than that of strontium-incorporated perovskites. Moreover, CO_2_ conversions in strontium-incorporated perovskites increased over time, which was not the case with CeNiO_3_. These findings infer that carbon gasification is promoted in an oxidative environment in strontium-incorporated perovskites, leading to no deactivation in these catalysts. The reaction mechanism and associated impact of Sr incorporation is further shown in Figure 8. The investigation of reduced La_2-x_Sr_x_NiO_4_ perovskite oxides by Rynkowski et al. [7] inferred that Sr incorporation in smaller amounts exhibited less-active but stable catalytic performance in comparison with strontium-free catalysts. The study of the role of LaNiO_3_ perovskites after partially substituting Ni and La for dry methane-reforming reaction revealed that La replacement with Sr leads to a loss of catalytic activity [54]. Furthermore, the comparison of findings in this work with already published literature (Table 2) revealed that the current conversions outperformed similar perovskites such as CeNiO_3_ [55] or similar combinations with varying A and/or B substitutions in ABO_3_ [56,57,58,59]. Since the main focus of this work was to demonstrate the deactivation factors as a function of catalytic stability of perovskites, it is obvious that the perovskites in this work showed lower deactivation factors than [55,59] despite using higher space velocity.

## 4. Materials and Methods

Nanocrystalline perovskites, i.e., Ce_x_Sr_1−x_NiO_3_ (x = 0.6–1), were prepared via the self-combustion method, and glycine and metallic nitrates were used as precursors. Initially, in 100 mL of deionized water, appropriate amount corresponding to x of hydrated cerium nitrate (Ce(NO_3_)_3_·nH_2_O, purchased from Sigma-Aldrich, St. Louis, MO, USA, with a purity of 99.9%), 1 mmole of nickel nitrate hexahydrate (Ni(NO_3_)_2_·6H_2_O purchased from Sigma-Aldrich with a purity of 99.9%), and appropriate amount corresponding to 1 − x of Sr(NO_3_)_2_ were dissolved. Then, glycine (purity of 99.5%) was added to metal nitrate solution as an ignition promoter by keeping the glyceine-to-metal-ions ratio close to unity. The mixtures were systematically stirred to obtain a homogenous gel-like solution by water elimination at 60–70 °C. Since the onset temperature for ignition reaction is ~250 °C, the gel was subjected to this temperature to produce powdered precursors that were eventually calcined under oxidative atmosphere at 700 °C for 6 h, leading to the formation of the perovskite structure. The nomenclature Ce_x_Sr_1−x_NiO_3_, where x varies between 0.6 and 1, was used in all cases just as standardization, as it is usual in the literature.

The thermogravimetric analysis (TGA), including differential curves (DTG) of the perovskite precursors, was recorded using a TGA/SDTA851e thermal analyzer (purchased from Mettler-Toledo, Switzerland). The samples were subjected to heating from room temperature to 1000 °C using a heating rate of 20 °C/min under inert atmosphere (by flowing nitrogen at 100 mL/min). The following characterizations were employed over calcined catalysts. X-ray diffraction profiles were recorded using a scan rate of 0.2°/min between 10 and 80° on an XRD-6000 diffractometer (purchased from Shimadzu, Columbia, MD, USA) equipped with monochromatic radiation of CuK (λ = 1.5406 Å). Braunner, Emmet, and Teller (BET) specific surface areas of the samples were measured on a NOVA2000 BET system (purchased from Quantachrome, Boynton Beach, FL, USA). Temperature-programmed oxidation (TPO) and reduction (TPR) profiles were conducted on an Autochem 2920 apparatus (purchased from Micromeritics, Norcross, GA, USA). A total of 30 mg of each sample was placed in a U-shape quartz tube. The sample was first pretreated under synthetic air flowing at 50 mL/min at 300 °C for 1 h followed by cooling it down to room temperature. In order to record TPR patterns, samples were subjected to reducing atmosphere by flowing 10% H_2_/Ar at 25 mL/min while the sample temperature was raised from room temperature to 900 °C at 10 °C/min. The TPO samples were pretreated in a way similar to TPR. TPO profiles were recorded for the spent catalysts post dry-reforming stability tests to identify carbon formed over the catalyst surface. The samples were subjected to oxidative environment by flowing 10% O_2_/He at 25 mL/min and raising the sample temperature from room temperature to 800 °C at 10 °C/min. The morphology of fresh and used perovskite catalysts was analyzed by transmission electron microscopy (TEM) using a JEM-210 microscope (purchased from JEOL, Tokyo, Japan) equipped with an accelerating voltage of 80 kV. The chemical compositions in the as-synthesized catalysts were measured by using inductively coupled plasma optical emission spectroscopy (ICP-OES ELAN 6100 purchased from Perkin Elmer, Waltham, MA, USA).

The dry methane-reforming reaction was conducted in a tubular fixed-bed reactor (i.d. 1 cm, catalytic bed length 3 mm) at 700 °C and 1 atm pressure. The feed gas, comprising methane and carbon dioxide (1:1) balanced with nitrogen, was flown at 70 mL/min with a space velocity of 84,000 mL g_cat._^−1^h^−1^. The catalysts were subjected to activation/reduction, prior to reaction, by flowing 10% H_2_ in nitrogen mixture (40 mL/min) at 700 °C for 2 h. After activation, the hydrogen mixture was replaced with helium to remove any leftover hydrogen and subsequently the reaction was carried out at 700 °C for over 7 h time-on-stream. The analysis of the reforming products and unconverted reactants was carried out using on-line gas chromatograph GC 3800 (purchased from VARIAN, Palo Alto, CA, USA) equipped with two columns (packed with Porapak N and 13X molecular sieves) and two thermal conductivity detectors. The results were reproducible with an error of ±5%. The conversions and molar ratios were calculated using following equations [60,61]:

(3) $${\mathrm{CH}}_{4}\;\mathrm{Converson}\;\left(\%\right)=100\times \frac{F_{\mathrm{CH}4,\mathrm{in}}-F_{\mathrm{CH}4,\;\mathrm{out}}}{F_{\mathrm{CH}4,\mathrm{in}}}$$

(4) $${\mathrm{CO}}_{2}\;\mathrm{Converson}\;\left(\%\right)=100\times \frac{F_{\mathrm{CO}2,\mathrm{in}}-F_{\mathrm{CO}2,\;\mathrm{out}}}{F_{\mathrm{CO}2,\mathrm{in}}}$$

(5) $$\frac{{\mathrm{nH}}_{2}}{\mathrm{nCO}}=\frac{F_{H2,\mathrm{out}}}{F_{\mathrm{CO},\mathrm{out}}}$$

## 5. Conclusions

This study demonstrated the catalytic performance results of strontium-incorporated Ce_x_Sr_1−x_NiO_3_ (x = 0.6–1) nanocrystalline perovskites for dry methane reforming. The extent of finely dispersed nanocrystalline particles over the surface of the catalysts, their reducibility, and their textural properties, including specific surface area, were determined by characterizing the catalysts prior to the reforming reaction. It was revealed that these factors significantly influenced the catalytic activity as well as durability. Strontium-free CeNiO_3_ perovskite demonstrated higher activity in terms of CH_4_ and CO_2_ conversions but showed deactivation over time-on-stream. Despite relatively lower conversions, strontium incorporated perovskites (Ce_0.8_Sr_0.2_NiO_3_ and Ce_0.6_Sr_0.4_NiO_3_) remained stable over the 7 h time-on-stream. The lower activity in strontium-incorporated perovskites was attributed to the coverage of active (nickel) sites with carbonates of strontium, which is also reported in the literature. The post-reforming characterizations of perovskites helped to investigate the deactivation cause. Despite finding active metal agglomeration or sintering over all catalysts post-reforming, the insignificant sintering was ruled out as major cause of deactivation substantiated with no activity loss for strontium-incorporated catalysts. This suggests that carbon formation during the reforming reaction was the main cause of deactivation. This was further confirmed by analyzing TEM and TPO results of spent perovskites. Stable and/or increasing CO_2_ conversions higher than CH_4_ conversions in strontium-incorporated perovskites facilitated carbon gasification, which prevented deactivation. These results presented the key role of the strontium incorporation in Ce_x_Sr_1−x_NiO_3_ (x = 0.6–1) perovskites to ensure stable catalytic performance for longer periods of time to avoid deactivation, a common challenge to cope with for nickel-based catalysts.

## Figures and Tables

**Table 1:** Textural properties and deactivation factors of Ce_x_Sr_1−x_NiO_3_(x = 0.6–1) perovskites.

Perovskites	Ni/Ce/Sr Content (%) ^a^		S_BET_ (m^2^/g)	Pore Volume (cm^3^/g)	Pore size (nm)	Deactivation Factor (%)	Coke (wt%) ^d^
	Fresh	Used					
CeNiO_3_	49.8/50.1/-	50.2/49.6/-	20.7	0.162	30.1	7.7 ^b^ (0.88) ^c^	9.1
Ce_0.8_Sr_0.2_NiO_3_	51.1/44.1/4.7	50.9/43.8/5.3	25.6	0.261	40.8	−62.6 ^b^ (1.71) ^c^	4.7
Ce_0.6_Sr_0.4_NiO_3_	50.7/32.1/17.2	51/31.1/17.8	17.3	0.164	38.1	−13.4 ^b^ (1.15) ^c^	2.1

^a^ Determined from ICP-OES. ^b^ Deactivation factor (D.F., %) = 100 × (CH_4_conversion_initial_ − CH_4_conversion_final_)/(CH_4_conversion_initial_). ^c^ D.F based on first-order deactivation, % = ln(1 − CH_4_conversion_final_)/ln(1 − CH_4_conversion_initial_). ^d^ Calculated from TPO data.

**Table 2:** Comparison of current work with previously reported work. DF refers to deactivation factor. TOS indicates time-on-stream.

Catalyst	Reaction Temp. (°C)/GHSV (L/h/g_cat_)	Highest CH_4_ Conversion (%)	%DF ^a^	TOS (h)	Ref.
CeNiO_3_	750/72	32	12.9	~26	[55]
La_0.6_Sr_0.4_NiO_3_	700/-	85	-	20	[56]
La_0.9_Sr_0.1_NiO_3_	700/18	70	3.6	8	[57]
LaNi_0.8_Fe_0.2_O_3_	800/13.7	65	7.7	35	[58]
La_0.5_Sr_0.5_NiO_3_	750/18	69	47.8	24	[59]
CeNiO_3_	700/84	55	7.7	8	This work
Ce_0.8_Sr_0.2_NiO_3_		22.5	−62.6		
Ce_0.6_Sr_0.4_NiO_3_		22.5	−13.4		

^a^ Deactivation Factor (D.F., %) = 100 × (CH_4_conversion_initial_ − CH_4_conversion_final_)/(CH_4_conversion_initial_).

## Data Availability

Not applicable.

## Supplementary Materials

The following supporting information can be downloaded, Table S1: Textural properties and deactivation factors of Ce_x_Sr_1−x_NiO_3_ (x = 0.6–1) perovskites.

## Sample Availability

Samples of the compounds Ce_x_Sr_1−x_NiO_3_ (x = 0.6–1) perovskites are available from the authors.

## References

[1] Wang Y., Yao L., Wang S., Mao D., Hu C. (2018). Low-temperature catalytic CO_2_ dry reforming of methane on Ni-based catalysts: A review. Fuel Process. Technol..

[2] Li H., He Y., Shen D., Cheng S., Wang J., Liu H., Xing C., Shan S., Lu C., Yang R. (2017). Design an in-situ reduction of Ni/C-SiO_2_ catalyst and new insight into pretreatment effect for CH_4_-CO_2_ reforming reaction. Int. J. Hydrogen Energy.

[3] Al-Fatesh A.S., Arafat Y., Atia H., Ibrahim A.A., Ha Q.L.M., Schneider M., M-Pohl M., Fakeeha A.H. (2017). CO_2_-reforming of methane to produce syngas over Co-Ni/SBA-15 catalyst: Effect of support modifiers (Mg, La and Sc) on catalytic stability. J. CO_2_ Util..

[4] Ibrahim A.A., Fakeeha A.H., Al-Fatesh A.S. (2014). Enhancing hydrogen production by dry reforming process with strontium promoter. Int. J. Hydrogen Energy.

[5] Li D., Lu M., Xu S., Chen C., Zhan Y., Jiang L. (2017). Preparation of supported Co catalysts from Co-Mg-Al layered double hydroxides for carbon dioxide reforming of methane. Int. J. Hydrogen Energy.

[6] Al-Fatesh A.S., Arafat Y., Ibrahim A.A., Atia H., Fakeeha A.H., Armbruster U., Abasaeed A.E., Frusteri F. (2018). Evaluation of Co-Ni/Sc-SBA–15 as a novel coke resistant catalyst for syngas production via CO_2_ reforming of methane. Appl. Catal. A Gen..

[7] Rynkowski J., Samulkiewicz P., Ladavos A.K., Pomonis P.J. (2004). Catalytic performance of reduced La_2−x_Sr_x_NiO_4_ perovskite-like oxides for CO_2_ reforming of CH_4_. Appl. Catal. A Gen..

[8] El Hassan N., Kaydouh M.N., Geagea H., El Zein H., Jabbour K., Casale S., El Zakhem H., Massiani P. (2016). Low temperature dry reforming of methane on rhodium and cobalt based catalysts: Active phase stabilization by confinement in mesoporous SBA-15. Appl. Catal. A Gen..

[9] Khan W.U., Li X., Baharudin L., Yip A.C.K. (2021). Copper-promoted cobalt/titaniananorod catalyst for CO hydrogenation to hydrocarbons. Catal. Lett..

[10] Khan W.U., Baharudin L., Choi J., Yip A.C.K. (2021). Recent progress in CO hydrogenation over bimetallic catalysts for higher alcohol synthesis. ChemCatChem.

[11] Khan W.U., Chen S.S., Tsang D.C.W., Hu X., Lam F.L.Y., Yip A.C.K. (2020). Catalytically active interfaces in titaniananorod-supported copper catalysts for CO oxidation. Nano Res..

[12] Horn R., Schlögl R. (2015). Methane Activation by Heterogeneous Catalysis. Catal. Lett..

[13] Rostrup-Nielsen J.R., Sehested J., Nørskov J.K. (2002). Hydrogen and synthesis gas by steam- and CO_2_ reforming. Adv. Catal..

[14] Kang D., Yu J., Ma W., Zheng M., He Y., Li P. (2019). Synthesis of Cu/Ni-La_0.7_Sr_0.3_Cr_0.5_Mn_0.5_O_3–δ_ and its catalytic performance on dry methane reforming. J. Rare Earth.

[15] Wang F., Han K., Yu W., Zhao L., Wang Y., Wang X., Yu H., Shi W. (2020). Low Temperature CO_2_ Reforming with Methane Reaction over CeO_2_-Modified Ni@SiO_2_ Catalysts. ACS Appl. Mater. Interfaces.

[16] Han B., Zhao L., Wang F., Xu L., Yu H., Cui Y., Zhang J., Shi W. (2020). Effect of calcination temperature on performance of Ni@SiO_2_ catalyst in methane dry reforming. Ind. Eng. Chem. Res..

[17] Chein R.-Y., Fung W.-Y. (2019). Syngas production via dry reforming of methane over CeO_2_ modified Ni/Al_2_O_3_ catalysts. Int. J. Hydrogen Energy.

[18] Ma Q., Guo L., Fang Y., Li H., Zhang J., Zhao T.-S., Yang G., Yoneyam Y., Tsubaki N. (2019). Combined methane dry reforming and methane partial oxidization for syngas production over high dispersion Ni based mesoporous catalyst. Fuel Process. Technol..

[19] Abdullah B., Ghani N.A.A., Vo D.-V.N. (2017). Recent advances in dry reforming of methane over Ni-based catalysts. J. Clean. Prod..

[20] Ali S., Khader M.M., Almarri M.J., Abdelmoneim A.G. (2020). Ni-based nano-catalysts for the dry reforming of methane. Catal. Today.

[21] Shen J., Reule A.A.C., Semagina N. (2019). Ni/MgAl_2_O_4_ catalyst for low-temperature oxidative dry methane reforming with CO_2_. Int. J. Hydrogen Energy.

[22] Wanga F., Hana B., Zhanga L., Xub L., Yuc H., Shi W. (2018). CO_2_ reforming with methane over small-sized Ni@SiO_2_ catalysts with unique features of sintering-free and low carbon. Appl. Catal. B Environ..

[23] Han K., Yu W., Xu L., Deng Z., Yu H., Wang F. (2021). Reducing carbon deposition and enhancing reaction stability by ceria for methane dry reforming over Ni@SiO_2_@CeO_2_ catalyst. Fuel.

[24] Charisiou N.D., Siakavelas G., Papageridis K.N., Baklavaridis A., Tzounis L., Avraam D.G., Goula M.A. (2016). Syngas production via the biogas dry reforming reaction over nickel supported on modified with CeO_2_ and/or La_2_O_3_ alumina catalysts. J. Nat. Gas Sci. Eng..

[25] Goula M.A., Charisiou N.D., Siakavelas G., Tzounis L., Tsiaoussis I., Panagiotopoulou P., Goula G., Yentekakis I.V. (2017). Syngas production via the biogas dry reforming reaction over Ni supported on zirconia modified with CeO_2_ or La_2_O_3_ catalysts. Int. J. Hydrogen Energy.

[26] Makri M.M., Vasiliades M.A., Petallidou K.C., Efstathiou A.M. (2016). Effect of support composition on the origin and reactivity of carbon formed during dry reforming of methane over 5 wt% Ni/Ce_1−x_MxO_2−δ_ (M= Zr^4+^, Pr^3+^) catalysts. Catal. Today.

[27] Summa P., Swirk K., Wierzbicki D., Motak M., Alxneit I., Rønning M., Da Costa P. (2021). Co-Precipitated Ni-Mg-Al Hydrotalcite-Derived Catalyst Promoted with Vanadium for CO_2_ Methanation. Molecules.

[28] Świrka K., Gálvez M.E., Motak M., Grzybek T., Rønning M., Da Costa P. (2018). Dry reforming of methane over Zr- and Y-modified Ni/Mg/Al double-layered Hydroxides. Catal. Commun..

[29] Świrk K., Gálvez M.E., Motak M., Grzybek T., Rønning M., Da Costa P. (2018). Yttrium promoted Ni-based double-layered hydroxides for dry methane Reforming. J. CO2 Util..

[30] Swirk K., Rønning M., Motak M., Beaunier P., Da Costa P., Grzybek T. (2019). Ce- and Y-Modified Double-Layered Hydroxides as Catalysts for Dry Reforming of Methane: On the Effect of Yttrium Promotion. Catalysts.

[31] Jacobson A.J. (2010). Materials for Solid Oxide Fuel Cells. Chem. Mater..

[32] Kim W.Y., Jang J.S., Ra E.C., Kim K.Y., Kim E.H., Lee J.S. (2019). Reduced perovskite LaNiO_3_ catalysts modified with Co and Mn for low coke formation in dry reforming of methane. Appl. Catal. A Gen..

[33] Wei T., Jia L., Zheng H., Chi B., Pu J., Li J. (2018). LaMnO_3_-based perovskite with in-situ exsolved Ni nanoparticles: A highly active, performance stable and coking resistant catalyst for CO_2_ dry reforming of CH_4_. Appl. Catal. A Gen..

[34] Yang Q., Liu G., Yuan Liu Y. (2018). Perovskite-type oxides as the catalyst precursors for preparing supported metallic nanocatalysts: A review. Ind. Eng. Chem. Res..

[35] Bhattar S., Abedin M.A., Kanitkar S., Spivey J.J. (2021). A review on dry reforming of methane over perovskite derived catalysts. Catal. Today.

[36] Khalesi A., Arandiyan H.R., Parvari M. (2008). Production of Syngas by CO_2_ Reforming on M_x_La_1−x_Ni_0_._3_Al_0_._7_O_3−d_ (M = Li, Na, K). Ind. Eng. Chem. Res..

[37] Pérez-Camacho M.N., Abu-Dahrieh J., Goguet A., Sun K., Rooney D. (2014). Self-cleaning perovskite-type catalysts for the dry reforming of methane. Chin. J. Catal..

[38] Ruocco C., Caprariis B.D., Palma V., Petrullo A., Ricca A., Scarsella M., Filippis P.D. (2019). Methane dry reforming on Ru perovskites, AZrRuO_3_: Influence of preparation method and substitution of A cation with alkaline earth metals. J. CO2 Util..

[39] Wang H., Dong X., Zhao T., Yu H., Li M. (2009). Dry reforming of methane over bimetallic Ni-Co catalyst prepared from La(Co_x_Ni_1-x_)_0.5_Fe_0.5_O_3_ perovskite precursor: Catalytic activity and coking resistance. Appl. Catal. B Environ..

[40] Wang F., Xu L., Shi W. (2016). Syngas production from CO_2_ reforming with methane over core-shell Ni@SiO_2_ catalysts. J. CO_2_ Util..

[41] Han B., Wang F., Zhang L., Wang Y., Fan W., Xu L., Yu H., Li Z. (2020). Syngas production from methane steam reforming and dry reforming reactions over sintering-resistant Ni@SiO_2_ catalyst. Res. Chem. Intermed..

[42] Dehghani F., Ayatollahi S., Bahadorikhalili S., Esmaeilpour M. (2020). Synthesis and Characterization of Mixed–Metal Oxide Nanoparticles (CeNiO_3_, CeZrO_4_, CeCaO_3_) and Application in Adsorption and Catalytic Oxidation–Decomposition of Asphaltenes with Different Chemical Structures. Pet. Chem..

[43] Harikrishnan M.P., Mary A.J.C., Bose A.C. (2020). Electrochemical performance of ANiO_3_ (A= La, Ce) perovskite oxide material and its device performance for supercapattery application. Electrochim. Acta.

[44] Chen J., He Z., Li G., An T., Shi H., Li Y. (2017). Visible-light-enhanced photothermocatalytic activity of ABO_3_-type perovskites for the decontamination of gaseous styrene. Appl. Catal. B Environ..

[45] Hu Q., Yue B., Shao H., Yang F., Wang J., Wang Y., Liu J. (2020). Facile syntheses of cerium-based CeMO_3_ (M = Co, Ni, Cu) perovskite nanomaterials for high-performance supercapacitor electrodes. J. Mater. Sci..

[46] García de la Cruz R.M., Falcón H., Peña M.A., Fierro J.L.G. (2001). Role of bulk and surface structures of La_1−x_Sr_x_NiO_3_ perovskite-type oxides in methane combustion. Appl. Catal. B Environ..

[47] Wang Y., Cui X., Li Y., Shu Z., Chen H., Shi J. (2013). A simple co-nanocasting method to synthesize high surface area mesoporous LaCoO_3_ oxides for CO and NO oxidations. Microporous Mesoporous Mater..

[48] Wei Y., Zhao Z., Jiao J., Liu J., Duan A., Jiang G. (2015). Facile synthesis of three-dimensionally ordered macroporous LaFeO_3_-supported gold nanoparticle catalysts with high catalytic activity and stability for soot combustion. Catal. Today.

[49] Sudhakaran M.S.P., Hossain M.M., Gnanasekaran G., Mok Y.S. (2019). Dry Reforming of Propane over γ-Al_2_O_3_ and Nickel Foam Supported Novel SrNiO_3_ Perovskite Catalyst. Catalysts.

[50] Pino L., Italiano C., Lagana M., Vita A., Recupero V. (2020). Kinetic study of the methane dry (CO_2_) reforming reaction over the Ce_0.70_La_0.20_Ni_0.10_O_2−δ_ catalyst. Catal. Sci. Technol..

[51] Ostrovskii N.M. (2005). Problems in the Study of Catalyst Deactivation Kinetics. Kinet. Catal..

[52] Moral A., Reyero I., Alfaro C., Bimbela F., Gandía L.M. (2018). Syngas production by means of biogas catalytic partial oxidation and dry reforming using Rh-based catalysts. Catal. Today.

[53] Verykios X. (2003). Mechanistic aspects of the reaction of CO_2_ reforming of methane over Rh/Al_2_O_3_ catalyst. Appl. Catal. A Gen..

[54] Choudhary V.R., Uphade B.S., Belhekar A.A. (1996). Oxidative Conversion of Methane to Syngas over LaNiO_3_ Perovskite with or without Simultaneous Steam and CO_2_ Reforming Reactions: Influence of Partial Substitution of La and Ni. J. Catal..

[55] Lima S.M., Assaf J.M., Peña M.A., Fierro J.L.G. (2006). Structural features of La_1−x_Ce_x_NiO_3_ mixed oxides and performance for the dry reforming of methane. Appl. Catal. A Gen..

[56] Valderrama G., Goldwasser M.R., de Navarro C.U., Tatibouët J.M., Barrault J., Batiot-Dupeyrat C., Martínez F. (2005). Dry reforming of methane over Ni perovskite type oxides. Catal. Today.

[57] Das S., Bhattar S., Liu L., Wang Z., Xi S., Spivey J.J., Kawi S. (2020). Effect of Partial Fe Substitution in La_0.9_Sr_0.1_NiO_3_ Perovskite-derived Catalysts on Reaction Mechanism of Methane Dry Reforming. ACS Catal..

[58] Komarala E.P., Komissarov I., Rosen B.A. (2020). Effect of Fe and Mn Substitution in LaNiO_3_ on Exsolution, Activity, and Stability for Methane Dry Reforming. Catalysts.

[59] Mousavi M., Pour A.N., Gholizadeh M., Mohammadi A., Shahri S.M.K. (2020). Dry Reforming of Methane by La_0.5_Sr_0.5_NiO_3_ Perovskite Oxides: Influence of Preparation Method on Performance and Structural Features of the Catalysts. J. Chem. Technol. Biotechnol..

[60] Voorhoeve R.J.H., Burton J.J., Garten R.L. (1977). 5—Perovskite-Related Oxides as Oxidation—Reduction Catalysts. Advanced Materials in Catalysis.

[61] Messaoudi H., Thomas S., Djaidja A., Slyemi S., Barama A. (2018). Study of La_x_NiO_y_ and La_x_NiO_y_/MgAl_2_O_4_ catalysts in dry reforming of methane. J. CO2 Util..

