# Supplementary material for: The Role of Strontium in CeNiO3 Nano-Crystalline Perovskites for Greenhouse Gas Mitigation to Produce Syngas
Source: Molecules. 2022 Jan 6;27(2):356. doi: 10.3390/molecules27020356 (PMC8779912; doi:10.3390/molecules27020356)
Supplement: Supplementary file 1 [file molecules-27-00356-s001.zip › molecules-1507584-supplementary.pdf]

molecules

Supplementary Information

The role of strontium in $CeNiO_3$ nano-crystalline perovskites for greenhouse gas mitigation to produce syngas

Naushad Ahmad[1,*], Manawwer Alam[1], Rizwan Wahab[2], Salim Manoharadas[3], Basel F. Alrayes[4] and Fahad A. Alharthi[1]

[1] Department of Chemistry, College of Science, King Saud University, P.O. Box 2454, Riyadh 11451, Kingdom of Saudi Arabia; anaushad@ksu.edu.sa; fharthi@ksu.edu.sa; malamiitd@gmail.com
[2] Department of Zoology, College of Science, King Saud University, P.O. Box 2454, Riyadh 11451, Kingdom of Saudi Arabia; rwahab@ksu.edu.sa
[3] Central Laboratory, Department of Botany and Microbiology, College of Science, King Saud University, P.O. Box 2454, Riyadh 11451, Saudi Arabia; smanoharadas@ksu.edu.sa
[4] Central Laboratory, College of Science, King Saud University,P.O. Box 2454, Riyadh-11451, Kingdom of Saudi Arabia; bfalrayes@ksu.edu.sa
* Correspondence: Correspondence: anaushad@ksu.edu.sa;naushaddrnaima@gmail.com

Table S1. Textural properties and deactivation factors of $Ce_xSr_{1-x}NiO_3$(x = 0.6-1) perovskites.

**Perovskites**	**T1 (°C)**	**T2 (°C)**	**T3 (°C)**
$CeNiO_3$	150	420	-
$Ce_{0.8}Sr_{0.2}NiO_3$	145	410	-
$Ce_{0.6}Sr_{0.4}NiO_3$	130	450	870
